# Plumbagin Enhances the Radiosensitivity of Tongue Squamous Cell Carcinoma Cells via Downregulating ATM

**DOI:** 10.1155/2021/8239984

**Published:** 2021-08-27

**Authors:** Shu-Ting Pan, Gan Huang, Qiaohong Wang, Jia-Xuan Qiu

**Affiliations:** Department of Oral and Maxillofacial Surgery, The First Affiliated Hospital of Nanchang University, Nanchang 330006, Jiangxi, China

## Abstract

This study was designed to investigate whether plumbagin (PL) could sensitize ionizing radiation (IR) in tongue squamous cell carcinoma (TSCC) cells and its possible mechanisms. Cell proliferation and combination index analysis was based on MTT and colony formation assay. Flow cytometry was applied to analyze the cell cycle distribution and apoptosis after the treatment of PL and/or IR. RT-PCR was used to examine the gene expression level of ataxia telangiegatasiata muted (ATM) and nuclear factor kappa beta (NF-κB) after various treatment groups. Western blot was used to examine the protein level of ATM and NF-κB as well as their phosphorylation level. PL enhances the cytotoxicity of IR in TSCC cells. Combination index was <1 which represents a synergistic effect. Combined PL and IR promoted G2/M arrest and apoptosis which could be reversed by ATM activator chloroquine phosphate. ATM and NF-κB were both inhibited by PL and IR combination. PL can efficiently enhance the radiosensitivity of TSCC cells by inducing G2/M arrest and apoptosis via downregulating ATM.

## 1. Introduction

The prevalent pathological type of tongue cancers is squamous cell carcinoma (TSCC) of which the incidence has been increasing since the 2000s [1]. TSCC has the characteristics of rapid development, early metastasis, and high lethal rate. Although there has been advancement in the comprehensive therapies, the patients still suffer from serious relapse and 50% of patients die within 5 years after diagnosis [2].

The existence of radiation resistance greatly hampers the treatment efficacy of cancer [3]. One of the main reasons is the enhanced DNA repair ability in cancer cells which may affect the cell cycle and apoptotic cell death. The ATM gene is the main transducer of the DNA damage response. ATM can identify DNA damage sites, initiate DNA damage response, and recruit downstream proteins to repair damaged DNA [4]. Except for ATM, nuclear factor kappa beta (NF-κB) is another important transducer of DNA damage [5]. Abnormal activation of NF-κB will lead to the radioresistance and relapse of human cancers [6].

In the past decades, natural products from herbs have emerged as potent anticancer strategies used either alone or in combination with chemotherapy or radiotherapy in order to overcome resistance [7]. Plumbagin (PL) is an important bioactive secondary metabolite isolated from the root of *Plumbago zeylanica L.* which possesses anticancer activities [8]. Previous studies from us and other groups have shown that PL regulates cell cycle, apoptosis, and autophagy in various cancer cells, such as hepatocellular carcinoma [9], breast cancer [10], and tongue cancer [11]. PL can also enhance the anticancer efficacy of chemotherapeutic drugs such as cisplatin and paclitaxel through modulation of p53 and MAPKs pathways [12–14]. However, the sensitizing effect of PL on radiation of TSCC is not clear. In the present study, we aimed to determine the radiosensitizing effect of PL on TSCC cells as well as the mechanisms.

## 2. Materials and Methods

### 2.1. Chemicals and Reagents

PL was bought from Sigma-Aldrich Co. (USA). DMEM/F-12 medium, DMSO, fetal bovine serum (FBS), MTT, crystal violet, PBS, BSA, RIPA buffer, protease inhibitor cocktail, and PVDF were purchased from Solarbio Co. (China). Primary antibodies against ATM (#92356S, mouse mAb), p-ATM (#13050S, rabbit mAb), NF-κB (#8242S, rabbit mAb), and p-NF-κB (#3039S, rabbit mAb) were bought from Cell Signaling Technology Inc. (USA). All these antibodies were diluted into 1 : 1000. Primary antibody against *β*-actin (#660009-1-lg, mouse mAb) was purchased from Proeintech Inc. (USA). The dilution rate was 1 : 5000. Horseradish peroxidase-conjugated affinipure goat anti-rabbit lgG (#SA00001-2) and goat anti-mouse lgG (#SA00001-1) were purchased from Proteintech Inc. (USA). All the secondary antibodies were diluted to 1 : 2000. KU-55933 (KU) and chloroquine phosphate (CQ) were bought from Selleck Co. (USA).

### 2.2. Cell Line and Culture Conditions

TSCC cell line SCC9 was donated by the laboratory of Wuhan University, and CAL27 was bought from the Cell Bank of the Chinese Academy of Sciences (China). Cells were cultured with the DMEM/F-12 medium containing 10% (v/v) FBS, 100 *μ*g/ml streptomycin, and 100 U/ml penicillin at 37°C in a 5% CO_2_/95% humidified incubator.

### 2.3. Irradiation

TSCC cells were irradiated in a Primus X-ray linear accelerator 6 MV (Siemens, Germany) with a 2 Gy/min absorption dose rate and a 3 cm irradiation depth. The radiation dose was set separately according to the needs of each experiment. The irradiation field covers the whole cell culture plate, and two solid-water blocks (2 cm thickness) were placed on the top and bottom of the Petri dish. Cells were then incubated after radiation for next experiments.

### 2.4. Cell Viability Assay

MTT was applied to examine the effect of PL and/or IR on cell viability. TSCC cells were seeded into 96-well plates at a density of 5 × 10^3^ for incubation. After 24 hr, the cells were treated with PL at 0.625–20 *μ*M and/or IR at 1–8 Gy for 12, 24, and 48 hr. Then, a volume of 10 *μ*L of MTT solution was added to each well, and the plates were incubated for a further 3 hr. The solution was then removed, and 100 *μ*L DMSO was added to dissolve the crystal. The optical density (OD) was measured at the wavelengths of 560 nm and 670 nm using a Synergy^TM^ H4 Hybrid microplate reader (BioTek, Inc., USA). Cell viability was calculated as a percentage of the control. Cell viability (%) = (OD treatment-OD blank)/(OD control-OD blank) × 100%. The median inhibitory concentration (IC_50_) was calculated from the growth inhibition curves fitted to the data using GraphPad Prism 5 software.

### 2.5. Combination Index Analysis

The combination effect of PL and IR on TSCC cells was evaluated using the combination index (CI) according to the median dose-effect analysis by Chou and Talalay [15]. We designed 16 dose-effect points in a nonconstant ratio manner (Table 1). Fractional inhibition (FA) = 1-fraction of surviving cells. The corresponding CI values were analyzed using the CompuSyn Software (ComboSyn, Inc., USA). CI < 1 represents a synergistic effect; CI = 1 represents an additive cytotoxicity; and CI > 1 represents an antagonistic effect.

### 2.6. Colony Formation Assay

TSCC cells were seeded in six-well plates at a density of 500 cells/well. After 24 hr incubation, cells were treated with IR or PL alone or PL (pretreated for 1 hr) + IR. After 2 weeks, the medium was removed and cells were washed with PBS, fixed with 4% paraformaldehyde, and stained with 0.1% crystal violet for 30 min. Colonies with more than 50 cells were scored and counted under the microscope.

### 2.7. Cell Cycle Distribution Analysis

TSCC cells were seeded in six-well plates at a density of 1 × 10^5^ cells/ml for attaching overnight. Then, cells were treated with IR or PL alone or PL (pretreated for 1 hr) + IR. After 24 hr, cells were trypsinized and fixed with cold 70% ethanol overnight. The Cell Cycle detection kit (KeyGEN BioTECH Co., China) containing PI and RNase was used to incubate the cells for 30 min. A total of 1 × 10^4^ events were subjected to cell cycle analysis by flow cytometry (BD™ LSR II Analyzer, USA).

### 2.8. Apoptosis Assay

TSCC cells were seeded in six-well plates at a density of 1 × 10^5^ cells/ml for attaching overnight. Then, cells were treated with IR or PL alone or PL (pretreated for 1 hr) + IR. After 24 hr, cells were trypsinized and washed twice with cold PBS. The Annexin V: PE/7-AAD apoptosis detection kit (BD Biosciences Inc., USA) containing annexin V: PE and 7-AAD was used to bind the cells for 15 min at room temperature. 1 × binding buffer (400 *μ*L) was added to each test tube, and the apoptosis number was quantified by flow cytometry (BD™ LSR II Analyzer, USA).

### 2.9. Real-Time Polymerase Chain Reaction

Total RNA was extracted from TSCC cells based on the protocol of TRIzol reagent. The collected RNA was used to synthesize cDNA with a reverse transcription kit (Takara, Japan). The reaction condition was 37°C for 15 min and 85°C for 15 s. For measuring the mRNA levels of ATM and NF-κB, real-time quantitative PCR was performed with the SYBR Premix Ex Taq kit (QIAGEN, Germany) in a DNA Engine Opticon 2 system (Bio-Rad, USA). The mRNA level of *β*-actin was used to standardize the mRNA levels. The reaction procedures were denaturation at 95°C for 5 min, 40 cycles at 95°C for 30 s, and 60°C for 45 s. The comparative CT method (ΔΔCT) was used to compute the relative variations in gene expression. The sequences of primers for ATM, NF-κB, and *β*-actin are provided as follows: *β*-actin: Forward-5′-CGTGGACATCC TAAAGACC-3′, Reverse-5′-ACATCTGCTGGAAGGTGGAC-3′; ATM: Forward-5′-CGTGCCAGAATGTGAACACC-3′, Reverse-5′-A GCCAATACTGGACTGGTGC-3′; and NF-κB: Forward-5′-AACAGCAGA TGGCCCATACCT-3′, Reverse-5′-ACGCTGAGGTCCATCTCCT TG-3′.

### 2.10. Western Blot Analysis

TSCC cells were seeded in six-well plates at a density of 1 × 10^5^ cells/ml for attaching overnight. Then, cells were treated with IR or PL alone or PL (pretreated for 1 hr) + IR. After 24 hr, cells were washed with PBS and lysed with RIPA buffer. Nuclear proteins were extracted using the nuclear protein extraction kit (Beyotime Co., China). Protein concentrations were measured by using the BCA protein assay kit (Beyotime Co., China). Equal amounts of protein samples were electrophoresed on 8–10% sodium dodecyl sulfate polyacrylamide gel electrophoresis (SDS-PAGE). Proteins were transferred onto an immobilon PVDF membrane at 100 V for 2 hr at 4°C. Membranes were probed with primary antibodies overnight at 4°C and then blotted with secondary antibodies for 2 hr. Super ECL Star (US Everbright, Inc.) and the UVP ChemStudio Imaging System (Analytik Jena, Germany) were employed to examine the electrochemiluminescence of indicated proteins.

### 2.11. Statistical Analysis

All the experimental data are presented as mean ± standard deviation (SD) in triplicates. Multiple comparisons were evaluated by one-way analysis of variance (ANOVA) followed by Tukey's post hoc test. GraphPad Prism 5 software was used for statistical analysis. *p* < 0.05 was considered statistically significant.

## 3. Results

### 3.1. PL Inhibits the Proliferation and Enhances the Radiosensitivity of TSCC Cell Lines

**Figure 1:**
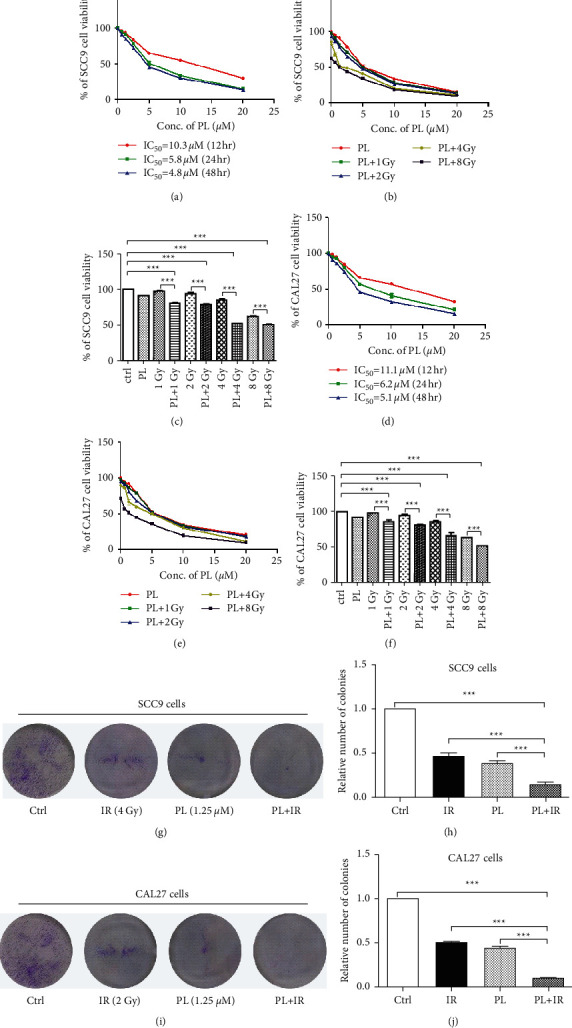
The effect of PL and/or IR on TSCC cell viability. (a, d) SCC9 and CAL27 cells were treated with 0.625–20 *μ*M PL for 12, 24, and 48 hr. (b, e) Combination treatment of various doses of PL and IR for 24 hr in SCC9 and CAL27 cells. (c, f) Histograms of the cell viability after the combination treatment of 1.25 *μ*M PL and 1–8 Gy IR for 24 hr. (g, i) SCC9 and CAL27 cells were treated with IR, PL, and IR + PL for 24 hr. (h, j) Colony formation quantification of TSCC cells with PL or combined with IR. ^*∗∗∗*^*p* < 0.001.

As shown in Figures 1(a) and 1(d), PL treatment decreased the viability of TSCC cells in a dose- and time-dependent manner. After 12, 24, and 48 hr treatment, the IC_50_ of PL in SCC9 cells was 10.3, 5.8, and 4.8 *μ*M; the IC_50_ of PL in CAL27 cells was 11.1, 6.2, and 5.1 *μ*M, respectively.

We then explored whether PL could enhance IR sensitivity of TSCC cells. We tested the combined effect of PL (1.25, 2.5, 5, and 10 *μ*M) with IR (1, 2, 4, and 8 Gy) on TSCC cells. As illustrated in Figures 1(b), 1(c), 1(e), and 1(f) and Table 1, the combination treatment significantly increased the IR sensitivity in TSCC cells. In addition, CI values showed that the combination of PL and IR exerted synergistic cytotoxic effects at all tested points in SCC9 cells and at most tested points in CAL27 cells (Table 1). Noteworthy, the combination of 1.25 *μ*M PL and 4 Gy IR exhibited the lowest CI value (CI = 0.626) in SCC9 cells and the combination of 1.25 *μ*M PL and 2 Gy IR exhibited the lowest CI value (CI = 0.692) in CAL27 cells which indicated the best synergistic effect.

Based on the aforementioned data, we selected the dose of 1.25 *μ*M PL and 4 Gy IR for SCC9 cells and 1.25 *μ*M PL and 2 Gy IR for CAL27 cells in the next experiments. Colony formation assay showed that the combination of PL and IR could significantly decrease the number of colonies by 44.81% in SCC9 cells and 55.82% in CAL27 cells, compared with IR single treatment (Figures 1(g)–1(j); ^*∗∗∗*^*p* < 0.001). These results showed that PL significantly radiosensitized the TSCC cells.

### 3.2. Combined PL and IR Promote G2/M Arrest in TSCC Cells

**Figure 2:**
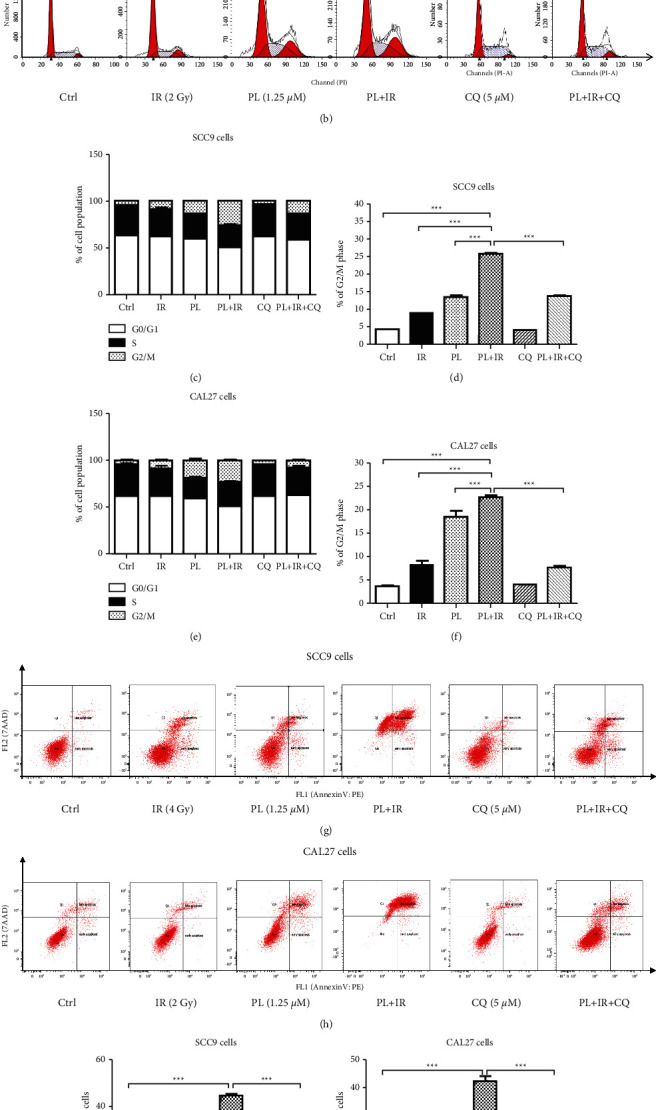
Combined PL and IR treatment promotes G2/M arrest and apoptosis in TSCC cells. (a) Representative flow cytometric plots of cell cycle distribution of SCC9 cells and (c-d) bar graph showing the percentage of TSCC cells in G0/G1, S, and G2/M phases after the treatment of 4 Gy IR, 1.25 *μ*M PL, and the combination of 4 Gy IR with 1.25 *μ*M PL for 24 hr. (b) Representative flow cytometric plots of cell cycle distribution of CAL27 cells and (e-f) bar graph showing the percentage of CAL27 cells in G0/G1, S, and G2/M phases after the treatment of 2 Gy IR, 1.25 *μ*M PL, and the combination of 2 Gy IR with 1.25 *μ*M PL for 24 hr. (g-h) Representative flow cytometric plots of apoptosis of SCC9 and CAL27 cells and (i-j) bar graph showing the percentage of total apoptosis in SCC9 and CAL27 cells after the treatment of IR, PL, and IR + PL for 24 hr. ^*∗∗∗*^*p* < 0.001.

To probe the mechanisms of synergism between PL and IR in TSCC cells, we first detected the cell cycle distribution by PI staining and flow cytometry. SCC9 cells were treated with 1.25 *μ*M PL and/or 4 Gy IR for 24 hr, while CAL27 cells were treated with 1.25 *μ*M PL and/or 2 Gy IR for 24 hr. In SCC9 cells, compared with the IR single group, the PL and IR combination group significantly increased the G2/M phase by 1.89-fold (Figures 2(a), 2(c), and 2(d); ^*∗∗∗*^*p* < 0.001). In CAL27 cells, compared with the IR single group, the PL and IR combination group significantly increased the G2/M phase by 1.76-fold (Figures 2(b), 2(e), and 2(f); ^*∗∗∗*^*p* < 0.001). Collectively, these results show that combination PL and IR treatment modulates cell cycle rendering TSCC cells less proliferative in a potent and synergistic manner.

### 3.3. PL Enhances IR-Induced Apoptosis in TSCC Cells

We next examined whether PL could affect the cellular apoptosis and whether apoptosis is a mechanism of synergism between PL and IR. SCC9 cells were treated with 1.25 *μ*M PL and/or 4 Gy IR for 24 hr, while CAL27 cells were treated with 1.25 *μ*M PL and/or 2 GyIR for 24 hr. As shown in Figures 2(g) and 2(h), PL and IR treatment alone could increase apoptosis. When PL and IR were combined together, the apoptosis levels were significantly increased by 3.65- and 2.84-fold comparing with the IR group, in separate SCC9 and CAL27 cells (Figures 2(i) and 2(j); ^*∗∗∗*^*p* < 0.001). Collectively, these results show that combination PL and IR treatment modulates apoptosis, rendering TSCC cells more apoptotic in a potent and synergistic manner.

### 3.4. PL Inhibits the IR-Induced Phosphorylation of ATM and NF-*κ*B in TSCC Cells

To probe the mechanism of radiosensitization effect of PL in TSCC, we used ATM activator CQ to examine the induction effect of cell cycle and apoptosis by PL and IR combination. Compared with PL + IR treatment, pretreatment of 5 *μ*M CQ for 1 hr decreased the G2/M phase population by 46.52% in SCC9 cells and 66.44% in CAL27 cells (Figures 2(a)–2(f)). While compared with PL + IR treatment, pretreatment of 5 *μ*M CQ for 1 hr decreased the apoptosis rate by 54.68% in SCC9 cells and 57.04% in CAL27 cells (Figures 2(g)–2(j)).

**Figure 3:**
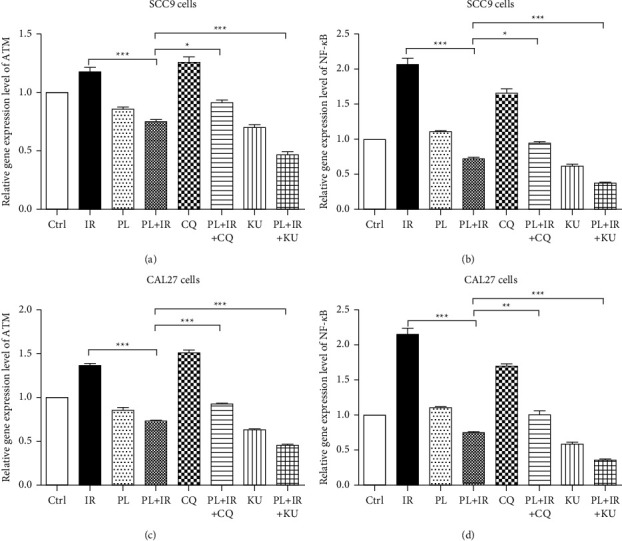
mRNA level of ATM and NF-κB after the pretreatment of ATM activator CQ or ATM inhibitor KU in TSCC cells. (a-b) Relative mRNA level of ATM and NF-κB in SCC9 cell after the treatment of 1.25 *μ*M PL and 4 Gy IR with or without pretreatment of 5 *μ*M CQ or 100 nM KU. (c-d) Relative mRNA level of ATM and NF-κB in CAL27 cell after the treatment of 1.25 *μ*M PL and 2 Gy IR with or without pretreatment of 5 *μ*M CQ or 100 nM KU. ^*∗*^*p* < 0.05, ^*∗∗*^*p* < 0.01, and ^*∗∗∗*^*p* < 0.001.

**Figure 4:**
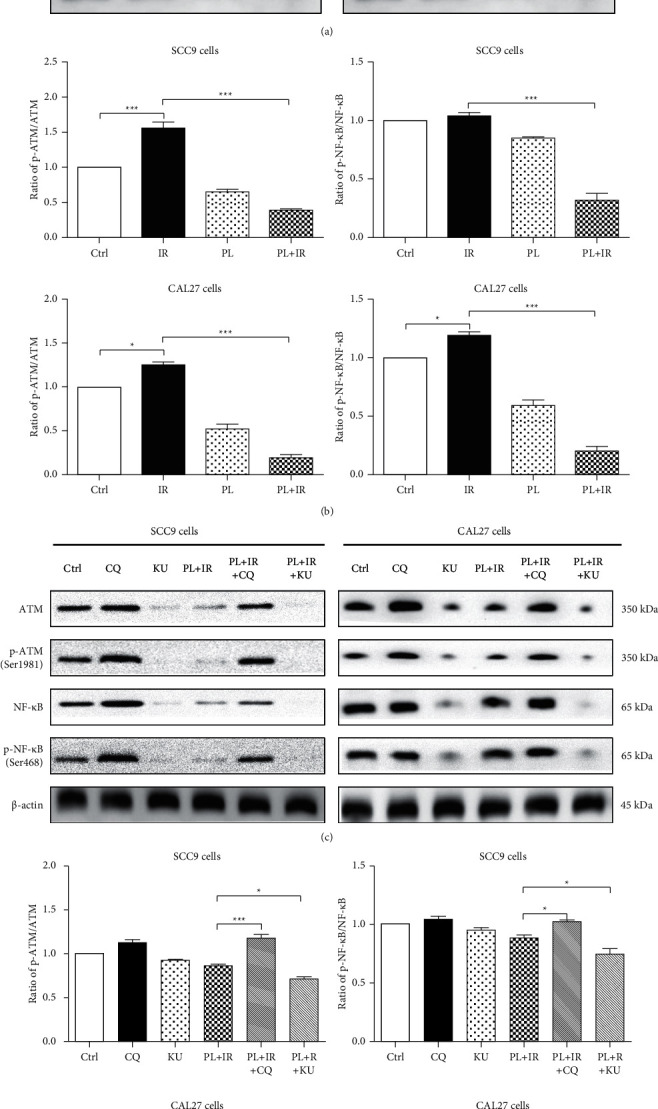
Combined PL and IR inhibit ATM signaling in TSCC cells. (a) Representative bolts of ATM, p-ATM, NF-κB, and p-NF-κB in SCC9 and CAL27 cells after the treatment of IR, PL, and IR + PL for 24 hr. (c) Representative bolts of ATM, p-ATM, NF-κB, and p-NF-κB in SCC9 and CAL27 cells after the treatment of CQ, KU, PL + IR, PL + IR + CQ, and PL + IR + KU for 24 hr. (b, d) Bar graphs showing the ratio of p-AMT/ATM and p-NF-κB/NF-κB when the cells were treated with various groups for 24 hr. ^*∗*^*p* < 0.05, ^*∗∗*^*p* < 0.01, and ^*∗∗∗*^*p* < 0.001.

Furthermore, RT-PCR and Western blot were used to examine the expression level of ATM and NF-κB in both cells. Comparing with the IR single group, the mRNA level of ATM was decreased by 35.98% and the mRNA level of NF-κB was decreased by 64.94% after the combined treatment of PL and IR in SCC9 cells (Figures 3(a) and 3(b); ^*∗∗∗*^*p* < 0.001); the mRNA level of ATM was decreased by 46.32% and the mRNA level of NF-κB was decreased by 65.17% after the combined treatment of PL and IR in CAL27 cells (Figures 3(c) and 3(d); ^*∗∗∗*^*p* < 0.001). The protein levels of ATM, p-ATM, NF-κB, and p-NF-κB were all increased after the treatment of IR. However, when IR combined with PL, these proteins were all downregulated (Figures 4(a) and 4(b); ^*∗∗∗*^*p* < 0.001). Furthermore, we applied ATM activator CQ and inhibitor KU to examine the exact modulation role of PL on ATM. mRNA level of ATM could efficiently be upregulated by 5 *μ*M CQ while downregulated by 100 nM KU. Noteworthy, modulation ATM significantly affected the mRNA level of NF-κB. CQ could elevate the level of NF-κB by 1.66-fold, and KU could decrease the level of NF-κB by 38.33% (Figure 3; ^*∗∗∗*^*p* < 0.001). This hints that ATM is a potent upstream of NF-κB. In addition, pretreatment of 5 *μ*M CQ for 1 hr reversed PL and IR combination-induced downregulation of p-ATM and p-NF-κB (Figures 4(c)and 4(d)).

Collectively, these results showed that PL significantly sensitized TSCC cells to radiation by inhibiting the IR-induced phosphorylation of ATM and NF-κB.

## 4. Discussion

TSCC is one of the most lethal cancer types in the head and neck region, and the five-year survival rate has not improved significantly [2]. Radiotherapy is an important method for treating TSCC by inducing DNA breaks [16].

PL is a naturally-derived active naphthoquinone which shows promising medicinal properties. Our group focuses on the anticancer property of PL for many years. The main findings are that PL could efficiently kill tongue cancer cells via cell cycle arrest, apoptosis, and autophagy induction through modulating PI3K/Akt/mTOR signaling. Ono et al. was the first to show that PL and cisplatin could synergistically enhance apoptosis in OSCC cells [14]. Similarly, in our recent research, the combination treatment of PL and cisplatin resulted in a synergistic inhibition of TSCC viability [13]. In the present study, we revealed that PL could enhance IR sensitivity in TSCC cells. The combination of 1.25 *μ*M PL and 4 Gy IR exhibited the lowest CI value in SCC9 cells, and the combination of 1.25 *μ*Μ PL and 2 Gy IR exhibited the lowest CI value in CAL27 cells which indicated the best synergistic effect. Due to the reduced dosage of PL and IR when combined together, it will be beneficial to minimize the side effects of PL and/or IR to the normal cells or tissues.

The radiation sensitivity of cancer cells changes according to the phase of the cell cycle. There is a resistance in the G0/G1 phase, and the resistance reaches the highest level in the late S phase. After entering the G2/M phase, the cell sensitivity increased again [16]. In our previous studies, PL could arrest TSCC cells at the G2/M phase in a concentration- and time-dependent manner. The expression level of Cdc2 and Cyclin B1 was decreased, while the expression level of p21, p27, and p53 was increased [11]. In this present study, we found that combined PL and IR treatment significantly promotes G2/M arrest which is an underlying mechanism of radiosensitization of PL in TSCC cells.

Agents targeting apoptosis would be expected to be used in combination with radiotherapy to delay tumor growth [17]. Palbociclib, the CDK4/6 inhibitor, can promote apoptosis of bladder cancer cells by inhibition of DNA repair following radiotherapy [18]. The Bcl-2 and Bcl-XL inhibitor S44563 could significantly enhance the sensitivity of small-cell lung cancer cells to radiation [19]. Our present research revealed that PL with the concentration of 1.25 *μ*M could greatly induce apoptosis to enhance the radiosensitivity of TSCC cells. In this study, we did not examine whether PL-induced apoptosis in TSCC cells was through the intrinsic or extrinsic pathway. Based on our previous data, both intrinsic and extrinsic pathways are involved in the PL-induced apoptosis in TSCC cells [11, 20]. Other groups reported that PL-induced apoptosis via the intrinsic mitochondrial pathway in non-small-cell lung cancer A549 cells [21]. PL could also enhance TRAIL-induced apoptosis in leukemic Kasumi-1 cells [22]. Collectively, the present research is the first to report the radiosensitizing effect of PL in TSCC cells via induction of G2/M arrest and apoptosis.

ATM belongs to the phosphoinositide 3-kinase family which involves in cell proliferation, survival, and metabolism. ATM can respond to radiation-induced DNA damage and launch downstream repair proteins and cell cycle checkpoints to render cancer cells survival, so inhibiting the expression of ATM can appropriately increase the sensitivity of cells to radiation [4, 23]. In the present study, the IR single treatment group increased the phosphorylation of ATM in TSCC cells. In the combined treatment of PL and IR, the level of p-ATM was decreased comparing with the IR group. Pretreatment of ATM activator CQ could reverse the induction effect of apoptosis and G2/M arrest by the PL and IR combination. In addition, CQ could reverse PL and IR combination-induced downregulation of p-ATM and p-NF-κB. ATM inhibitors will probably block NF-κB activation that is induced by radiotherapy [24]. NF-κB can regulate the survival and malignancy of many cancers including tongue cancer [25, 26]. Our research found that ATM inhibitor KU could decrease the mRNA level of NF-κB in TSCC cells. IR single treatment activated NF-κB in both mRNA and protein level, while PL combined with IR significantly decreased the expression level of NF-κB. CQ reversed PL-induced NF-κB inhibition. All these demonstrate that PL can enhance radiosensitivity by inhibiting ATM signaling.

In conclusion, we provide evidence that the combination of PL and IR efficiently facilitates the G2/M arrest and apoptotic activity by downregulating ATM in TSCC cells. PL was a potent radiosensitizer. Although preclinical and clinical investigations are still necessary, combined treatment of PL and IR seems to be a potentially attractive modality for tongue cancer.

## Figures and Tables

**Table 1:** CI of various concentrations of PL combined with four doses of IR.

SCC9		FA				CI			
PL (*μ*M)	IR (Gy) 0	1	2	4	8	1	2	4	8
0	—	0.067	0.108	0.289	0.398	—	—	—	—
1.25	0.084	0.188	0.322	0.479	0.497	0.901	0.708	0.626	0.967
2.5	0.217	0.287	0.349	0.511	0.561	0.987	0.967	0.771	0.961
5	0.488	0.498	0.521	0.587	0.655	0.949	0.977	0.946	0.979
10	0.661	0.711	0.732	0.791	0.812	0.976	0.948	0.816	0.847

CAL27		FA				CI			
PL (*μ*M)	IR(Gy) 0	1	2	4	8	1	2	4	8
0	—	0.065	0.109	0.352	0.412	—	—	—	—
1.25	0.078	0.179	0.341	0.398	0.505	0.944	0.692	0.882	1.058
2.5	0.210	0.291	0.345	0.512	0.612	0.967	0.997	0.816	0.902
5	0.473	0.501	0.526	0.652	0.668	0.929	0.968	0.802	1.007
10	0.651	0.712	0.745	0.801	0.813	0.956	0.899	0.792	0.882

## Data Availability

The data used to support the findings of this study are included within the article.
